# Differing responses in milk composition from introducing rapeseed and naked oats to conventional and organic dairy diets

**DOI:** 10.1038/s41598-019-44567-8

**Published:** 2019-05-31

**Authors:** Gillian Butler, Sokratis Stergiadis, Eleni Chatzidimitriou, Enrica Franceschin, Hannah R. Davis, Carlo Leifert, Håvard Steinshamn

**Affiliations:** 1 School of Natural and Environmental Science, Newcastle University, Newcastle upon Tyne, NE1 7RU UK; 2 School of Agriculture, Policy and Development, University of Reading, Reading, RG6 6AR UK; 3 Mérieux NutriSciences, Via Fratta, 25, 31023, Resana, Italy; 4 Centre for Organics Research, Southern Cross University, Lismore, NSW Australia; 5 Norwegian Institute of Bioeconomy Research, Department of Grassland and Livestock, Høgskoleveien, Norway

**Keywords:** Systems biology, Diseases

## Abstract

Dairy products are often considered challenging for health due to their saturated fatty acid content, yet they also provide beneficial nutrients, some unique to ruminants. The degree of fat saturation is influenced by cows’ diets; grazing pasture enhances unsaturated fatty acids in milk compared with conserved forages. These benefits can be partially mimicked by feeding oilseeds and here we consider the impact on milk composition in a 2 × 2 trial, feeding rapeseed to both conventional and organic cows, finding very differing lipid metabolism in the 4 experimental groups. For milk fat, benefits of organic rather than conventional management (+39% PUFA, +24% long chain omega-3 and +12% conjugated linoleic acid (CLA)) appear complementary to those from feeding rape (+43% MUFA, +10% PUFA, +40% CLA), combining to produce milk 16% lower SFA and higher in MUFA (43%), PUFA (55%) and CLA (59%). Organic and rape feeding provide less omega-3 PUFA than the conventional and control diets, yet contrary to expectations, together they almost doubled (+94%) the omega-3 concentration in milk, implying a 3.8 fold increase in net transfer from diet into milk. Organic and rape feeding also gave lower trace-elements and antioxidants in milk. Greater understanding of these phenomena might enhance the sustainability of dairying.

## Introduction

There is a lot of interest in nutritional contribution of milk and dairy products to our diet, possibly due to diverging attitudes towards the relationship between their consumption and our health. On one hand they are high in saturated fatty acids (SFA) which might challenge health but they also supply many unsaturated fatty acids and antioxidants, beneficial to our health^1^ and are a valuable source of quality protein, calcium and iodine^2,3^. Modern, western diets have many weaknesses but whilst advice to cut total fat consumption will help reduce calories and the most harmful saturated fatty acids (C12:0, C14:0 and C16:0), it will also diminish lipid soluble beneficial nutrients like vitamins A and E as well as omega-3 fatty acids (n-3), already deficient in our diets^4^. An alternative approach would be to alter the composition of the food on offer, reducing the proportion of SFA whilst enhancing the supply of monounsaturated (MUFA) and polyunsaturated fatty acids (PUFA) along with other beneficial nutrients.

The nutrient composition of animal products, including milk and dairy, is a reflection on what we feed our animals. Although ruminants like cattle and sheep see major modification of many nutrients by microbial action in the rumen, especially for fatty acids^5^, we still find substantial variations in milk and meat quality from differing animal diets, often reflecting seasonal changes or system differences in feeding practices^6–10^. For example, a survey on the fatty acid profiles in UK retail milk^6^ found more conjugated linoleic acid (CLA, +32%), n-3 (+60%) and total PUFA (+24%) in organic compared with conventional milk. This study also identified 6% less SFA, 13% more MUFA and PUFA (30% more n-3 and 42% more CLA) in summer compared with milk purchased in the winter, when cows are likely to be housed and fed conserved silage rather than grazing. We can mitigate these system and seasonal differences in milk composition to some extent, by offering feeds high in MUFA or PUFA such as whole soya beans, sunflower, rape or linseed to mimic the grazing missing for housed cattle^11,12^. Another approach might be to replace regular feeds like more common cereals or by-products feeds with those of greater lipid and PUFA content, such as naked oats^13^ or distillers grains^14^, although this has not be exploited to the same extent as oilseeds. Whilst it is recognised that introducing such feeds into dairy diets will enhance the fatty acid profiles of the resulting milk, it is unclear what impact this might have on other constituents, like antioxidants or minerals in the milk.

Here we describe a controlled feeding trial involving 2 dairy herds, run in parallel on the same farm. This was conducted in winter on silage diets, to assess the impact of and interactions between a) management system (conventional vs organic^15^) and b) supplementing their control diets with rapeseed, on the concentration of a number of nutritionally relevant constituents in milk. All procedures were acceptable to internal ethical review, in accordance with EU Directive 2010/63/EU for animal experiments and approved by the Animal Welfare and Ethical Review Body at Newcastle University.

## Materials and Method

This 2 × 2 study was carried out in parallel dairy herds of, year-round calving, Holstein–Friesian cows at Newcastle University’s Nafferton farm, as described by Stergiadis, *et al*.^11^. The 2 Nafferton dairy herds, established in 2006, are independent although under common supervision; one managed to organic standards^15^, allowing a system comparison without the bias of differing stockmanship or environmental conditions. This experiment consisted of two separate but simultaneous trials, one in the conventional and one in the organic herd, both of a nested design with cows in each herd randomly allocated to treatment groups, blocked for lactation number, days in milk, milk yield, gross milk composition (fat, protein and lactose) and somatic cell count (SCC) based on the last recording prior to selection. Milk samples, proportionate to yield, were taken from individual cows twice in 24 h (between 05.30–07.00 at morning and 13.30–15.00 at afternoon milking) the day before the trial started (A) and repeated on day 14, 35, 56 and 70 (B, C, D and E respectively) days after introducing treatment diets, with morning and evening samples mixed before being stored at −20 °C until analysis. Milk yield and results of analysis on the samples collected on date A, on the eve of the trial, are presented in Table S1. Both herds were loose housed with wheat straw bedding added daily and feed offered once a day as a mixed ration (TMR), with additional commercial concentrate feed provided in the parlour during milking.

Standards for organic dairying relate to housing, access to grazing, health and fertility treatments but also cover feeding. At least 60% of dry matter intake (DMI) has to be forage, hence concentrate intakes on organic farms are often lower than conventional herds. However, to reduce unnecessary differences between the herds for this study, concentrate supplementation was comparable – possibly aimed below the average for conventional farms but higher than typical organic herds. Forage proportion of DMI averaged 63% across trials and treatments for the organic herd, compared with 60% for the conventional cows. Although forage type is not defined by organic regulations, in the absence of nitrogen fertilizer, reliance on fixation by legumes results in more red and white clover in organic forages.

### Feeding

Table 1 gives diet detail for the 4 groups of cow for the duration of the trial. This study aimed to monitor the impact on milk production and quality from changing the mixed diet for conventionally and organically managed cows, by replacing i) 2 kg per cow per day of the standard pre-mix with rolled rapeseed and ii) all wheat and barley in the pre-mix with naked oats; assessing the impact of the same batch of supplements against contrasting basal diets. Logistics of simultaneously milking multiple groups of cows prevented a fully nested 2 × 2 × 2 format, so the main study involved a 2 × 2 design for 10 weeks, to consider basic management (conventional v organic) with and without rapeseed supplementation. Forty cows from each herd were allocated into two balanced groups, on average 147 days from calving for the conventional herd and 178 days for organic cows, at the start of the trial. The impact of oats was assessed as a changeover, with naked oats replacing wheat and barley in the diet for all 4 groups between days 36 and 56, before reverting to the original diet for the final 14 days.

**Table 1:** Diet details (kg feed per cow per day) throughout the trial.

Management and supplementation	Conventional herd		Organic herd	
	Control	Rape	Control	Rape
Period 1, milk sampling B & C				
Grass (clover) silage	41.7		49.0	
Whole crop wheat			3.8	
Compounded feed	3.9		4.09	
Premix: comprising of:	4.6		4.75	
Rolled wheat	1.7	0.8	0.5	0.5
Rolled barley			2.3	1.8
Crimped beans	1.1	0.8	1.9	0.5
Extracted soya bean meal	1.0	0.4		
Rolled rapeseed		1.7		1.8
Rolled naked oats				
Molasses	0.6	0.5		
Minerals	0.1	0.1	0.1	0.1
Period 2, milk sampling D				
Grass (clover) silage	38.3		44.7	
Whole crop wheat			4.8	
Compounded feed	4.1		4.5	
Premix, comprising of:	4.6		4.3	
Rolled wheat				
Rolled barley				
Crimped beans	0.9	0.7	1.9	0.4
Extracted soya bean meal	0.7	0.2		
Rolled rapeseed		1.4		1.7
Rolled naked oats	2.2	1.6	2.3	2.1
Molasses	0.6	0.6		
Minerals	0.1	0.1	0.1	0.1
Period 3, Milk sampling E				
Grass silage	37.4		41.8	
Whole crop wheat			4.2	
Compounded feed	4.2		4.7	
Premix, comprising of:	4.3		4.2	
Rolled wheat	1.6	0.75	2.1	1.7
Rolled barley			0.4	0.4
Crimped beans	1.1	0.8	1.7	0.4
Extracted soya bean meal	1.0	0.4		
Rolled rapeseed		1.7		1.7
Rolled naked oats				
Molasses	0.6	0.5		
Minerals	0.1	0.1	0.1	0.1

### Feed sampling and analysis

Samples of in-parlour concentrate feeds, premix ingredients, premixes, silages and mixed-rations were collected weekly and stored at −20 °C until preparation and chemical analysis. Three pooled samples of each feed for each sub-group were freeze dried before being sent for chemical analysis at DairyOne laboratory, New York, US. Frozen samples of all silages were also sent to Eurofins laboratory (Wolverhampton) for routine near infra-red spectrophotometry (NIR) prediction of fermentation characteristics and nutritional composition. Results from chemical analysis and NIR prediction of silage quality are presented in Tables S2 & S3 respectively.

### Milk yield and basic composition

Milk yields were automatically logged during milking and subsamples for all sampling dates were submitted to the National Milk Record laboratory (Harrogate, UK) for standard analyses of fat, protein and lactose using a Milkoscan FT 6000 (Foss Electric, Hillerod, Denmark) and for SCC using a Fossomatic instrument (Foss Electric). Energy corrected milk yield (ECM) was calculated by ECM = [0.327 × yield (kg/d)] + [12.86 × fat (kg/d)] + [7.65 × protein (kg/d)]^16^. Results of analysis on samples collected on date A, before introducing rapeseed, are presented in Table S1

### Milk analysis for antioxidant and trace elements

Trace element analysis was carried out by commercial lab Thompson and Joseph by Inductively Coupled Plasma Emission Mass Spectrometry (ICP-MS), quantifying copper, iodine, manganese, molybdenum, selenium and zinc. Extraction and assessment of antioxidants was based on Lietz, *et al*.^17^ with slight modifications; a) detection and quantification of retinol, β-carotene, lutein and zeaxanthin used a diode-array detector at 325 nm and 450 nm respectively and b) fluorescence detector was used for α-tocopherol at excitation and emission wavelengths at 285 nm and 325 nm respectively, with echinenone as the internal standard. Results of analysis on samples collected on date A presented in Table S4

### Blood sampling and analysis

Two heparinised venous blood sample were collected from each cow after morning milking on the last day of the trial. One was used to assess their metabolic profile (Albumin, Globulin, Total Protein, γ-Glutamyltransferase (GGT), Urea, β-hydroxybutyrate (βHB), Magnesium, Phosphate) by the farm’s vet practice and the other was centrifuged to collect plasma for fatty acid determination.

### Fatty acid determination in feed, milk and plasma

The procedures to measure the fatty acid profiles in feeds, milk and plasma were as outlined for experiment 2 in Stergiadis, *et al*.^11^. The fatty acids concentrations in dietary ingredients, premixes and mixed rations are presented in Tables S5 (for forages and novel feeds) & S6 (for the premixed concentrates and TMR) and the fatty acid profiles of the milk samples collected on date A are presented in Table S7a–d.

### Calculated dietary FA intakes and PUFA, LA and ALA recovery from diet into milk

Dietary intakes (g) of individual FA and FA groups from different experimental groups were calculated as follows. Reported daily fatty acid intakes (g)^18^ = fat content of feed (g/kg) × (% of individual FA or FA group in total FA/100). Net recovery of PUFA, LA and ALA from feed to milk was calculated as PUFA, LA or ALA in milk (g)/PUFA, LA or ALA intake (g), where: PUFA, LA and ALA in milk (g) = milk yield (g) × [milk fat content (g/100 g milk)/100)] × [PUFA,LA or ALA (g/100 g total FA) × 0.933 (correction factor representing % of FA in total milk fat^19^)/100)]. LA/ALA intake = feed provided (g DM) × [feed lipid content (g/100 g DM)/100] × % of [PUFA/LA/ALA (g/100 g total FA in feed)/100].

### Statistical analysis

All statistical analyses were performed in R^20^ and residual normality assessed using the qqnorm function^21^, with no data showing deviation from normality. Analyses of variance (ANOVA) used linear mixed-effects models (code: lme)^22^, with management (conventional/organic), dietary treatment (control/rape) and sampling date (A, B, C, D & E) as the main fixed factors and individual cows as random factor. Differences between dates and interactions between the main factors, where significant, were assessed using Tukey’s honest significant difference test (P < 0.05), based on a mixed-effects model.

Principal component analyses using vegan in R (D.pca <- rda(FAD1.1), plot(D.pca, display = “sites”, main = “FA Profile”), plot(D.pca, display = “species”, main = “ Each FA”)) was used to assess the concentrations of the 72 individual fatty acids in 319 samples of milk, over the 4 sample dates during the trial – identified by management x treatment groups for the cows. This was repeated separately with results from the last sampling date, pooling results for the concentrations of nutritionally relevant individual FA, in both milk and blood, against treatment groups.

## Results

The vast quantity of data collected in this study (in excess of 45 000 individual results or calculated values) runs the risk of obscuring key novel messages if all results are presented and discussed, hence this section of the paper focuses on particularly striking or novel findings. Other results are only touched on or summarised, although supplementary tables give more detail, with comprehensive results of mean feed, milk and blood composition with AVOVA p-values for differences between management, rape feeding and sampling date and their interactions. Unless stated otherwise, any differences mentioned here and in discussion are significant (p < 0.05), many highly so (p < 0.0001).

Table S8 presents results for milk output and basic composition during the study. The overall yield of energy corrected milk averaged 29.3 kg per cow per day (sem = 0.36) throughout the trial and did not differ (p > 0.05) between the organic and conventional herds, treatment groups or sampling dates. There was also a consistent daily yield of fat (1.08 kg/cow/day) and protein (0.85 kg/cow/day) between the experimental groups, during the trial, although the content of milk fat, protein and lactose did vary. Cows fed rape had lower milk protein (3.22% for control and 3.06% for those on rape) and higher milk lactose (4.38% for control v 4.52% for those on rape) and cows under conventional management produced milk with higher milk fat content compared with organically managed cows - 4.13% vs 3.88% respectively.

The next sub-sections cover the highly significant and substantial differences in milk mineral content and fat composition, with respect to the fatty acid and antioxidant profiles – influenced by both management system and rape supplementation, with some also changing over time, particularly reflecting the introduction of naked oats at date D. Plasma fatty acid composition assessed at the end of the study, also showed differences explained by management groups and rape supplementation – details discussed later. Table S9 with results from the metabolic profiles shows that, whilst average blood urea and phosphorus levels were higher for organic cows, differences were slight (5.2 vs 4.8 and 2.1 vs 1.9 mmol per litre with p < 0.05 and 0.0001 respectively) and values for all cows fell within the normal reference range. This also applied to albumin, globulin, total protein, GGT, βHB and magnesium concentrations - none of which varied between herds or treatment groups.

### The influence of management and rape on milk mineral and antioxidant profiles

The content of trace elements and antioxidants in milk is presented in Table S10. In the absence of routine mineral and vitamin supplementation, milk from organic cows was lower in some trace elements; exceptions being zinc (which did not differ) and molybdenum (higher in organic milk). Iodine concentration in organic milk only reached 53% that of conventional milk, selenium 56% and manganese at 86% (Table S10). Impact of management was also seen for the vitamin and carotenoid levels throughout the trial (presented in Table S10), with concentrations for organic milk only reaching 25–74% of those in conventional milk. What was less expected, however, were significantly lower concentrations of β-carotene, lutein, total carotenoids and manganese in milk taken at the initial sampling (date A) from cows allocated to rape treatment, before any rape was fed (Table S4)– making interpreting subsequent results challenging.

### The influence of management and rape feeding on milk fatty acid profiles

Results for the concentrations of all individual FA and FA groups in milk and how they were influenced by the main factors are presented in Table S11a–d, with results for key nutritionally relevant fatty acids also in Table 2. Of the 72 individual fatty acids quantified, only 12 did not differ between management systems, only 4 were not significantly altered by introducing rape to the diet and 19 showed significant interactions between these 2 factors.

**Table 2:** Mean concentrations of nutritionally relevant fatty acid in milk (g/kg total fatty acid) for management and treatment groups during the trial. Details of all milk FA are presented in supplementary material - Table S13 a–d. Key: Conven = conventional management, P-values: ***p < 0.001, **p < 0.01; *p < 0.05, t = 0.5 < p < 0.1, ns: p > 0.1. Fatty acid abbreviations: VA = vaccenic acid C18:1t11, OA = oleic acid C18:1c9, LA = linoleic acid C18:2 c9,12, ALA = alpha-linolenic acid C18:3c9,12,15, CLA9 = conjugated linoleic acid C18:2c9t11, SFA = saturated fatty acids, MUFA = monounsaturated fatty acids, PUFA = polyunsaturated fatty acids, n-3 = omega-3 fatty acids, n-6 = omega-6 fatty acids.

Fatty acid	Management		Supplement		sem	ANOVA p-values		
	conven	organic	control	rape		M	S	MxS
	n = 160	n = 159	n = 159	n = 160	n = 319			
C12:0	35.5	30.3	39.3	26.5	0.47	***	***	ns
C14:0	112	107	123	96	1.0	**	***	ns
C16:0	301	281	346	236	3.5	***	***	ns
C18:0	120	127	92	155	2.0	t	***	t
VA	10.1	12.0	9.0	13.1	0.18	***	***	ns
OA	213	211	170	254	2.7	ns	***	ns
LA	10.5	18.9	14.8	14.6	0.27	***	ns	**
ALA	5.5	9.3	7.0	7.8	0.13	***	***	ns
CLA9	5.1	5.7	4.5	6.3	0.09	*	***	ns
**Calculated values**								
SFA	677	663	724	617	3.5	*	***	ns
MUFA	287	287	235	338	3.3	ns	***	ns
PUFA	36.3	50.4	41.3	45.4	0.49	***	***	*
n3	10.3	14.1	11.7	12.7	0.14	***	***	ns
n6	15.7	24.8	19.8	20.6	0.30	***	ns	**
n3:n6	0.66	0.58	0.61	0.63	0.006	***	ns	**
n6:n3	1.52	1.79	1.69	1.62	0.017	***	t	***
Odd chain	22.2	22.4	25.7	18.9	0.22	ns	***	ns
Long chain n-3	1.73	2.11	1.96	1.88	0.019	***	ns	*

Both ‘treatments’ (organic management and rape supplementation) reduced total SFA in milk, as well as the concentrations of the 3 most harmful saturated fatty acids (C12:0, C14:0 and C16:0) and enhanced the concentrations of total PUFA, and individual FA considered beneficial. Together, the concentrations of C12:0, C14:0 and C16:0 were 7% lower in milk from the organic herd compared with conventional milk (418 vs 449 g/kg total fatty acid) and rape feeding depressed their combined concentration by 42%, compared with milk from the control diets (358 vs 509 g/kg total fatty acid). Overall PUFA concentration was 39% higher in organic compared with non-organic milk (50.4 vs 36.3 g/kg total fatty acid) and the rape fed group produced milk 10% higher in PUFA than cows on the control diets (45.4 vs 41.3 g/kg total fatty acid). For the individual MUFA and PUFA, milk from cows under organic management was higher in: ALA (+69%, 9.3 vs 5.5 g/kg total fatty acid), long chain n-3 (+22%, 2.1 vs1.7 g/kg total fatty acid) and total n-3 (+37%, 14.1 vs vs 10.3 g/kg total fatty acid) as well as LA, (+80%, 18.9 vs 10.1 g/kg total fatty acid) CLA9 (+12%, 5.7 vs 5.1 g/kg total fatty acid) and VA (+19%, 12.0 vs 10.1 g/kg total fatty acid), compared with milk from cows under conventional management. Supplementing diets with rapeseed also elevated the concentration of many but not all these FA. Milk from cows fed rapeseed had more ALA (+11%, 7.8 vs 7.0 g/kg total fatty acid), total n-3 (+9%, 12.7 vs 11.7 g/kg total fatty acid), CLA9 (+40%, 6.3 vs 4.5 g/kg total fatty acid) and VA (+46%, 13.1 vs 9.0 g/kg total fatty acid) but did not differ in the concentration of LA or long chain n-3 compared to milk from control diets.

Most of the statistically significant interactions between the main factors appear for minor fatty acids (together making up less than 1.8% of total FA), although they also include linoleic acid (LA), total n-6 and butyric acid. Rape feeding made little difference to the butyric acid in milk from conventional cows (27.3 vs 26.1 g/kg total FA, for control and rape feeding respectively) but raised its concentrations for the organic herd (30.3 g/kg total FA for control cows and 34.0 g/kg for those on rape supplementation). In some respects, the opposite was seen for LA. Concentrations in milk from conventional cows were elevated by rape feeding (10.0 vs 11.1 g/kg total, for control and rape respectively) whereas for the organic herd, rape depressed the concentration of LA (19.7 vs 18.1 g/kg total FA, for control and rape respectively). A very similar interaction was seen for total n-6 PUFA since LA is the dominant n-6. A significant interaction was also seen for the concentrations of long chain n-3 FA – the lower level in conventional milk was not altered by feeding rape (1.71 vs 1.75 g/kg total FA for control and rape respectively) whereas rape diets depressed concentration of long-chain n-3 in milk from organic cows (2.22 vs 2.01 g/kg total FA for control and rape respectively).

### Results from redundancy analysis on milk fatty acids

**Figure 1:**
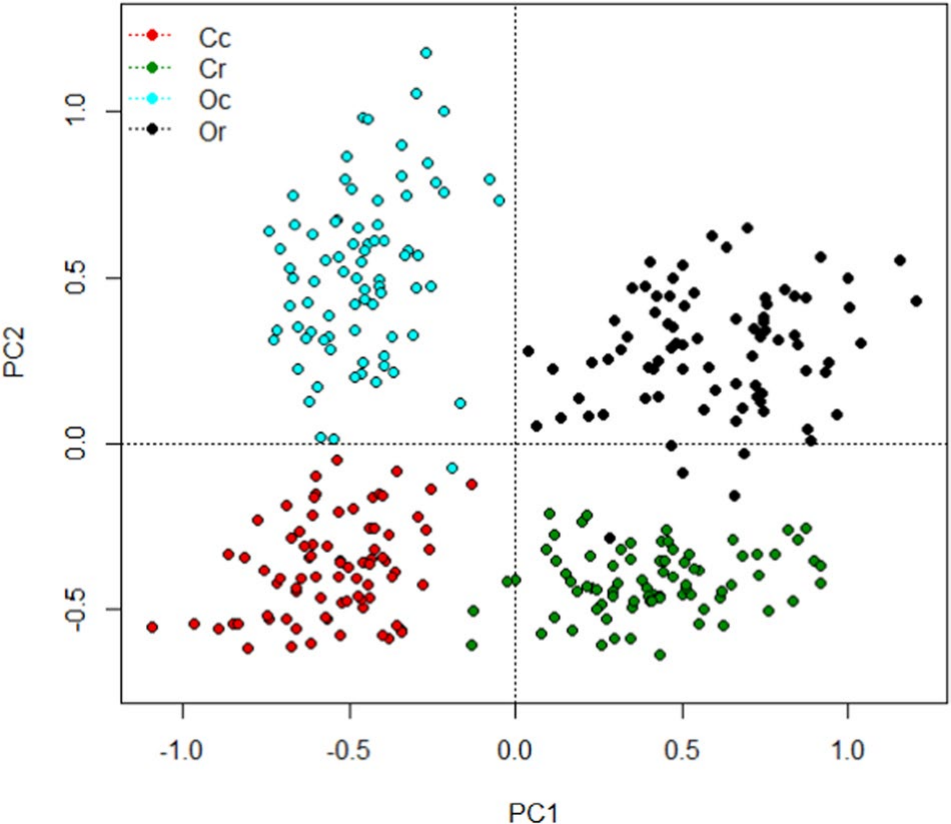
Bi-plot from principle component analysis based on individual fatty acid concentrations in milk collected on dates B, C, D and E, for individual cows specified by their management-by-treatment group. PC1 explains 41% and PC2 17% of variation in fatty acid profiles from Eigen values. Cc = conventional herd on control diets, Cr = conventional herd on rape diets, Oc = organic herd on control diets and Or = organic herd on rape diets.

Principal Component Analysis (PCA) identified a considerable spread in milk FA profiles within each ‘management-by-treatment’ group over the course of the study yet, despite this, there is very clear differentiation between the groups – almost without overlap, in the bi-plot depicted in Fig. 1. Generally, control v rape treatments differentiated either side of PC1 axis (responsible for 41% of variation) whereas the conventional v organic comparison was explained by PC2 (responsible for 17% of variation), with Eigenvalues of unconstrained axis of 30 and 12 respectively. The range within 3 of the management-by-treatment groups (Cc, Cr and Or) was greater along PC1 (especially for Cr), whereas variation in milk from Oc cows were more variable along PC2.

### Fatty acid intake and transfer into milk

**Figure 2:**
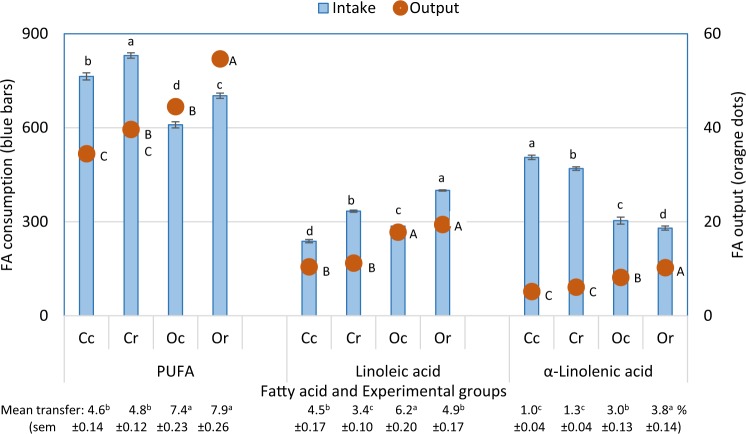
Intake (blue bars) and milk output (orange dots) of polyunsaturated fatty acid (PUFA), linoleic acid and alpha linolenic acid (g per cow per day) and mean net transfer rates (%) from diet to milk (error bars are standard errors of means). Cc = conventional herd on control diets, Cr = conventional herd on rape diets, Oc = organic herd on control diets and Or = organic herd on rape diets. For each fatty acid or groups of fatty acids, bars or mean values for transfer efficiency with the same lower case letter and dots with the same uppercase letter do not differ significantly (p > 0.05), according to Tukey’s honestly significant difference test.

Total lipid intake differed between management and treatment groups; conventional diets supplied more lipid than those fed to the organic cows (1506 g v 1317 g per cow per day) and on average both control diets were boosted by 585 g of lipid per cow per day with rape supplementation (control = 1118 g and rape = 1704 g per cow per day). Figure 2 shows the consequence of these differences in mean lipid consumption on LA, α-linolenic acid (ALA) and PUFA intake between groups. Cows in each treatment group also differed in their efficiency/efficacy of transferring these PUFA from diet into milk (p < 0.0001), resulting in differences in FA output into milk (p < 0.001). Figure 2 shows net transfer efficiency is higher (p < 0.0001) from the organic compared with conventional diets (for PUFA, (4.7% from conventional and 7.7% from organic) LA (4.0 v 5.6%) and ALA (1.2 v 3.4%)). Rape supplementation did not directly influence total PUFA transfer, possibly because the transfer of LA from diet to milk was reduced, whereas ALA transfer was enhanced, on rape compared with control diets (5.4 v 4.2% and 2.0 v 2.6% respectively). However, transfer of ALA shows a significant (p < 0.05) interaction between management and supplementation, which can be seen in Fig. 2. Feeding rape to organic cows enhanced the transfer of ALA from diet into milk (3.0% for control cows and 3.8% for those getting rape) whereas this is not seen with conventional cows who showed transfer rates of only 1.0 and 1.3% respectively - not significantly different.

### Comparing milk and blood composition

**Figure 3:**
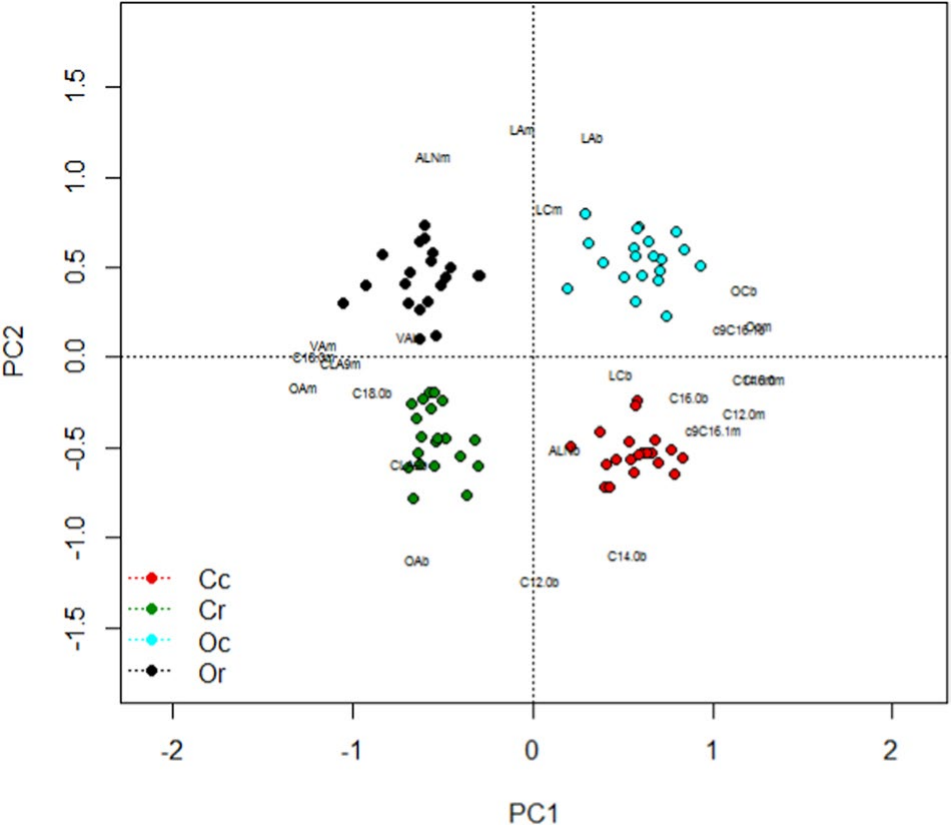
PCA bi-plot with key fatty acid concentrations in milk and blood sampled at date E identified for individual cows specified by their management-by-treatment groups. PC1 explains 48% and PC2 25% of variation fatty acid profiles from Eigen values. Fatty acid abbreviations: ALA = alpha-linolenic acid C18:3c9,12,15, CLA9 = conjugated linoleic acid C18:2c9t11, LA = linoleic acid C18:2 c9,12, LC = long chain omega-3 fatty acids (>C18), OA = oleic acid C18:1c9, OC = odd chain fatty acids VA = vaccenic acid C18:1t11. Blood concentrations shown as ‘b’ and milk as ‘m’. Cc = conventional herd on control diets, Cr = conventional herd on rape diets, Oc = organic herd on control diets and Or = organic herd on rape diets.

At the end of the trial, on the last sampling date there were anomalies comparing FA concentrations in plasma with those in milk, especially for many nutritionally relevant fatty acids – driven by both management and rape supplementation. Similar to the profile of milk FA throughout the trial depicted in Fig. 1, the PCA bi-plot comparing the concentrations of key FA in plasma and milk (Fig. 3) shows clear differentiation of cows into the 4 clusters – associated with the 4 treatment groups. For many key FA the concentrations detected in plasma and milk fall in close proximity, in the same RDA quadrant. These include C14:0, C16:0, C16:1c9, vaccenic acid (VA, C18:1t11), LA, total omega-6 (n-6) and the ratio of n-6:n-3, sum of odd chain FA and SFA. On the other hand, for some FA, the concentrations identified for plasma and milk fall in different chart quadrants – concentrations in milk are not clearly linked to levels circulating in the body. This group comprises of C12:0, oleic acid (OA, C18:1 c9) and total MUFA, conjugated linoleic acid (CLA9, C18:2c9t11), alpha linolenic acid (ALA, C19:3c9,12,15), PUFA, total n-3 and long chain n-3. Looking at the actual FA concentration in milk and blood (Table 3) for some but not all FA, the pattern of change driven by management and rape feeding was consistent between the 2 sets of results. Both blood and milk concentrations of C12:0, C14:0, OA and MUFA were lower for organic cows, whilst C17:0, LA, PUFA, sum of odd chain FA and the ratio of n-6:n-3 were higher in milk and blood of the organic cows. Rape feeding elevated blood and milk concentrations of C18:0, VA, CLA9, OA, and MUFA and depressed C14:0, C15:0, C16:0, C17:0, C16:1 (c9 & t9), C17:1, docosapentaenoic acid (DPA), SFA and odd chain FA relative to control cows. In contrast, for some FA, concentrations in milk and blood follow opposite responses. Under organic management, cows had less ALA, eicosapentaenoic acid (EPA) and total n-3 in plasma yet produced milk with higher concentration. Similarly, cows fed rape had lower plasma C20:3n-3, PUFA, n-3 and n-6 concentrations but elevated their concentrations in milk.

**Table 3:** Relative concentrations of fatty acids in plasma and corresponding values for milk (g/total fatty acid) at the end of the trial – date E. Mean values shown for management (conventional v organic) and supplementation (control v added rapeseed) with p-values for these factors and their interactions.

Fatty acids	Results for blood								Results for milk							
	Management (M)		Supplement (S)			ANOVA p-values			Management (M)		Supplement (S)			ANOVA p-values		
	Conven	Organic	Control	Rape	Sem	M	S	MxS	Conven	Organic	Control	Rape	sem	M	S	MxS
C12:0	1.01	0.18	0.61	0.59	0.051	***	ns	ns	34.8	29.9	39.9	24.8	1.04	***	***	ns
C14:0	4.7	2.7	4.2	3.2	0.14	***	***	*	109	105	124	90.6	2.1	*	***	ns
C15:0	4.9	5.6	5.8	4.7	0.09	***	***	ns	10.3	10.6	12.3	8.6	0.23	t	***	ns
C16:0	128	126	134	120	1.2	ns	***	ns	283	273	341	215	7.6	t	***	ns
t9C16:1	2.9	2.9	3.3	2.5	0.06	ns	***	ns	3.0	2.7	3.0	2.8	0.04	***	**	ns
c9C16:0	5.3	5.4	6.3	4.3	0.14	ns	***	*	15.0	13.2	16.7	11.4	0.40	***	***	ns
C17:0	8.8	11.4	12.1	8.2	0.28	***	***	***	4.3	5.1	5.3	4.0	0.09	***	***	ns
C17:1	2.4	1.5	2.2	1.6	0.07	***	***	ns	1.7	1.6	1.9	1.4	0.04	ns	***	ns
C18:0	253	250	244	259	1.4	ns	***	ns	129	131	94.9	164	4.4	ns	***	*
VA	6.9	7.3	6.5	7.7	0.16	ns	***	ns	11.0	11.7	8.8	14.0	0.38	ns	***	ns
OA	155	92.5	104	1423	4.3	***	***	ns	227	212	170	269	6.1	**	***	ns
LA	245	315	290	270	4.6	***	***	ns	11.4	20.1	15.7	15.8	0.55	***	ns	**
ALA	49.8	45.7	48.5	47.0	0.72	**	ns	***	5.9	9.0	6.7	8.2	0.22	***	***	ns
CLA9	1.47	1.25	1.21	1.51	0.036	***	***	*	5.6	5.6	4.3	6.8	0.20	ns	***	ns
C20:3 n-6	25.8	31.3	32.4	24.7	0.03	***	***	ns	0.53	0.87	0.8	0.6	0.01	***	***	t
C20:3 n-3	18.8	20.4	20.4	18.8	0.37	*	*	ns	0.25	0.31	0.1	0.4	0.02	***	***	ns
EPA	13.6	10.6	12.4	11.7	0.34	***	ns	*	0.48	0.59	0.6	0.5	0.01	***	***	ns
DPA	15.9	16.6	17.2	15.2	0.29	ns	***	ns	0.83	1.01	1.0	0.8	0.02	***	***	t
DHA	1.9	2.3	2.4	1.9	0.09	*	**	ns	0.08	0.07	0.08	0.07	0.003	ns	t	ns
**Calculated values**																
SFA	405	401	406	300	1.4	ns	*	ns	657	660	724	593	8.0	ns	***	ns
MUFA	203	139	149	193	4.6	***	***	ns	304	289	235	358	7.6	*	***	ns
PUFA	392	460	444	408	4.8	***	***	ns	38.8	51.5	41.9	48.4	0.96	***	***	*
n-3	104	97.5	104	97.2	1.1	**	**	**	1.1	1.3	1.1	1.3	0.02	***	***	ns
n-6	279	353	330	302	5.0	***	***	ns	1.7	2.6	2.1	2.2	0.06	***	*	***
n3:n6	0.37	0.28	0.32	0.33	0.007	***	ns	*	0.64	0.52	0.57	0.59	0.010	***	t	***
n6:n3	2.71	3.66	3.25	3.12	0.071	***	ns	*	1.58	1.97	1.84	1.71	0.03	***	**	***
Oddchain	18.5	20.2	22.1	16.5	0.36	***	***	***	21.1	22.0	25.4	17.7	0.47	**	***	ns
Long n3	50.6	50.0	52.8	47.8	0.73	ns	**	ns	1.7	2.0	1.9	1.9	0.03	***	ns	t

Key: Conven = conventional management, *P*-values = ***p < 0.001, **p < 0.01; *p < 0.05, t = p < 0.1, ns = p > 0.1.

Fatty acid abbreviations: VA = vaccenic acid C18:1t11, OA = oleic acid C18:1c9, LA = linoleic acid C18:2 c9,12, ALA = alpha-linolenic acid C18:3c9,12,15, CLA9 = conjugated linoleic acid C18:2c9t11, EPA = eicosapentaenoic acid C20:5 n-3, DHA = docosahexaenoic acid C22:6n-3, DPA = docosapentaenoic acid C22:5n-3, SFA = saturated fatty acids, MUFA = monounsaturated fatty acids, PUFA = polyunsaturated fatty acids, n-3 = omega-3 fatty acids, n-6 = omega-6 fatty acids.

### Influence of replacing wheat and barley with naked oats

Comparing results for date D after feeding naked oats for 14 days with those from dates C and E (when wheat and barley were fed) identified significant changes in milk composition, although in most cases, changes were relatively small in magnitude, compared with those driven by other factors. See Tables S12 for trace elements and antioxidants and S13a-d for fatty acids.

**Figure 4:**
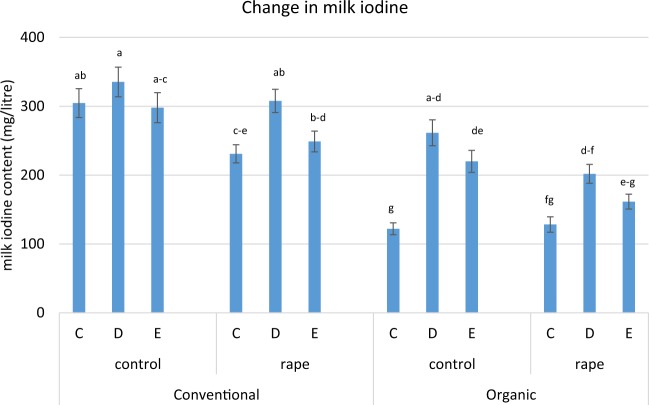
Changes in mean milk iodine concentration with the introduction of naked oats to the diet, between dates C (wheat), D (oats) and E (wheat) (error bars represent standard errors of means for each group). Bars with the same letter do not differ significantly (p > 0.05), according to Tukey’s honestly significant difference test.

On the whole, concentrations of individual short and medium chain FA (<C16) were between 2–10% higher with oat feeding, contributing to a small but significant increase of 1% in total SFA (66.7 vs 67.3%). The largest proportional changes in FA were reductions in the concentrations of a number of nutritionally relevant FA: VA, (−12%), ALA (−11%), n-3 (−9%) and CLA9, (−9%). Total MUFA was depressed by 2% and PUFA by 3%. The switch to oats also depressed β-carotene and hence total carotenoids in milk by 8% but elevated retinol by 4%. The most striking impact due to diet changes from wheat to naked oats relates to the iodine content of the milk, depicted in Fig. 4. No other trace elements were influenced by feeding oats (Table S12) whereas, between date C and date D, overall milk iodine content rose by 41%, with milk from organic cows increasing from an average of 125 to 232 mg/litre.

## Discussion

This study shows substantial influences on nutritionally relevant milk composition parameters driven by organic management and rapeseed supplementation, with many interactions between these 2 factors. However, the study was conducted on a semi-commercial farm without provision to measure feed intakes by individual cows, limiting replication for diet and hence fatty acid intakes and their transfer into milk. However, the large number of substantial differences in milk composition between treatment groups is stark, indicating important knowledge gaps that need exploring. Whilst some relationships identified here, between individual fatty acid intake by cows and output in milk, are well recognised and expected based on current knowledge, other findings are novel and more surprising. Results of feed, blood and milk analysis suggest very different lipid metabolism in cows (and/or their rumen microbiota) within the 4 management-by-treatment groups studied with, in many cases, those influenced by the basal diet being complimentary to those from lipid supplementation. The RDA approach adopted here is frequently used in ecology comparing species biodiversity in differing habitats, so is an ideal tool to assess variation in the profile of milk fatty acids produced under differing systems. Both bi-plots generated clearly cluster the 4 treatment groups into distinct and differing quadrants – all 4 ‘systems’ produced milk (and plasma profiles) with a distinct FA balance.

The ratio of forage to concentrate feeds in dairy diets is known to strongly influence milk fat secretion and composition^23,24^. However, despite similar ratios of forage and concentrates in diets for both management systems (60% forage for conventional and 63% for organic cows), we see confirmation of higher concentrations of many beneficial fatty acids including ALA, total n-3 and long chain n-3, along with less of the potentially damaging FA in organic milk^6,8,25,26^. We also saw the expected, preferential changes in milk fat composition from oilseed supplementation^11,12^.

Under organic management, milk SFA concentration was 14.1 g per kg total FA lower than in milk from the conventionally managed cows and coincidently, this appeared to be a direct ‘exchange’ with an extra 14.1 g PUFA per kg total FA of total FA. In addition to SFA being replace with PUFA, there also appeared to be further substitution between the individual saturated fatty acids, since the combined reduction in concentration of the harmful C12:0, C14:0 and C16:0 in organic milk was more than twice as great as the total SFA reduction, 31 g per kg total FA less than in conventional milk. Under this experiment with relatively high levels of LA in the concentrates fed to organic cows, the increase in milk PUFA was dominated by a combination of LA (60%) and ALA (27%), although there were also contributions from more CLA9 (4%) and long chain n-3 (3%).

Changes in milk fat composition brought about by supplementary rape appear to be complementary to those induced by organic management. Rape invoked a much greater reduction of SFA (−107 g/kg total FA) than the organic management, which was almost matched with an elevation in MUFA (+102 g/kg total FA), perhaps not a surprise since over 60% of the lipid in this rapeseed was MUFA (details in Table S5). Again there is a disproportionate reduction in the most harmful C12:0, C14:0 and C16:0 (together, 150 g less per kg total FA) with substitution by less damaging SFA as well as MUFA. On the other hand, the impact from feeding rape on individual and combined PUFA concentrations was weaker than observed for organic management, again, not a surprise in view of the FA profile of the rape diets. The noticeable exception with respect to individual milk PUFA was the elevation in CLA9 content, contributing almost half of the extra 4 g PUFA/kg total FA from the rape diets. This is likely to be explained by isomerisation of the enhanced dietary OA, by the rumen microbiota to create VA, as outlined by Jenkins *et al*.^27^ which was subsequently desaturated in the mammary gland to create CLA9^28,29^. This theory is supported by the higher concentrations of VA found in milk (+46%) from cows fed rape, compared to milk from control diets.

As it turns out, the higher levels of PUFA and n-3 found in the organic milk in this study were not due to greater n-3 PUFA supply in organic diets, as might be suspected, since the opposite was found. Comparing diet and milk composition, reveals one of the novel finding from this study - that the 2 groups of organic cows actually consumed 28% less total PUFA (636 g v 519 g per cow per day) and 58% less n-3 (484 g v 306 g per cow per day) than cows under conventional management, yet daily PUFA and n-3 output in milk from organic cows was 34% and 33% higher respectively, compare with conventional cows (Fig. 2). This suggests substantial differences in rumen and/or post-rumen fatty acid metabolism. The higher rates of PUFA and n-3 *transfer* into organic milk calculated here (4.7% and 1.2% for conventional cows and 7.7% and 3.4% for organic cows, respectively) might be explained by higher rumen turnover or rate of digesta leaving the rumen, reducing the extent of hydrogenation. It is possible that feeds included in organic dairy diets do have lower PUFA hydrogenation as a result of the combined effect of a) white clover silage having lower retention in the rumen (and hence hydrogenation) compared with grass silages^30^ along with b) suppressed lipolysis (and hence subsequent hydrogenation) under the influence of the enzyme polyphenol oxidase (PPO) in their red clover silage^31^. A similar, but weaker, impact was seen with n-3, when rolled rapeseeds replaced other feeds in the diets. This reduced ALA intake by 10% across both herds (406 g ALA per cow per day for control cows and 373 g ALA per cow per day for the rape group), yet the cows fed rape had a 23% higher ALA output in milk - again suggesting changes in the proportion of these dietary PUFA escaping intact from the rumen, without complete hydrogenation. Again, perhaps this can be explained since rapeseeds are rich in condensed tannins^32,33^, known to protect feeds from rumen degradation^33^.

However, if we consider the FA concentrations in the blood of these cows, perhaps this questions these theories - if rumen hydrogenation was lower under the influence of organic and rape diets, why do plasma FA profiles not reflect this, with elevated concentrations of circulating PUFA and n-3? The impact questioning these theories is particularly noticeable for ALA and n-3. Substituting rape into the conventional diet reduced plasma concentrations of ALA and n-3. Organic management also gave lower concentrations, compared to cows on the conventional control diet - although in this case, not changed by the inclusion of rape. Plasma concentrations of these fatty acids appear to mirror the amounts consumed from the diet, rather than dictate their rate of secretion into milk. Both organic management and rape supplementation incrementally increase ALA and n-3 concentrations in milk, despite lower intakes and plasma concentrations. There might well be differences in rumen hydrogenation as suggested by so many other studies^30,34^ but comparing the concentrations of FA in plasma with their output in milk from this study, implies other differences also exist. These changes must occur further along the lipid metabolism pathway after the absorption of dietary fatty acids into the blood stream and before milk secretion in the udder. They appear to be enhanced by both organic feeding regimes and the inclusion of rapeseed in the diet.

Whilst both organic management and rapeseed supplementation achieved the stated objectives of this study of improving milk fatty acid profiles with respect to consumer health, the other elements of milk quality considered here, did not have such positive outcomes. Overall, concentrations of trace elements and antioxidants also fluctuated with these experimental factors; levels of antioxidants decreased during the study but with considerable variations between the conventional and organic herds and with rape supplementation. Organic milk was lower than conventional milk in most trace elements and vitamins considered, largely as a result a lack of supplementation for the organic herd. Another potential explanation could be the lower vitamin E, β -carotene and lutein content of whole crop cereals and legumes (included in the organic diet) compared with grasses^35–37^. Organic silage was from grass clover and pure red clover swards, possibly with less antioxidants than the grass-based conventional silage. Since iodine and selenium play an important role in vitamin metabolism^38^ the relatively low levels of both elements in organic milk may be another explanation towards lower absorption and passage of vitamins and antioxidants into organic, compared with conventional milk.

Antioxidants concentrations in milk from control cows (not fed rape) decreased during the trial, suggesting a depletion of reserves accumulated from summer grazing. Ellis, *et al*.^39^, reported vitamin A concentrations in UK organic and conventional milk decreased during housing. Cows generally get vitamin A, its carotenoid precursors and vitamin E from fresh forages however, these oxidise over time, reducing their concentration in silage or hay^40^. Since this study was conducted in winter, antioxidant content in silage was probably relatively low, although not assessed. As well as this depletion over time, rape feeding also appeared to suppress antioxidant secretion into milk. Although concentrations of β-carotene and total carotenoids rose (not significantly) during the study period, other antioxidants in milk diminished, as with non-rape supplemented cows.

Trace elements were also higher in conventional milk, with the exception of zinc (not significantly different) and molybdenum, which was unsurprising given that only the conventional herd received routine mineral supplementation. Milk is an important source of iodine in our diet^2^, especially in the absence of salt fortification and the low levels of milk iodine from organic cows found here, confirms earlier work^8^. These findings triggered a change in attitude within organic sector, responding to likely deficiency in dairy cows as a consequence of a lack of iodine supplementation. Since this study in 2012, 2 actions have combined to potentially narrow the difference in iodine content between organic and conventional milk. More organic dairy farms are supplementing diets with iodine^41^ and the European Food Safety Authority has reduced the permitted maximum level of iodine in dairy diets from 5 to 2 mg/kg, as of 2015 (Regulation 2015/861). Organic Milk Suppliers cooperative reported in April 2017 that supermarket organic milk has comparable iodine concentrations to conventional milk.

The concentration of copper and molybdenum in milk decreased during the study - to a greater extent for cows fed rape. The concentration of other trace elements in milk from both herds increased during the trial (not always significantly), however to a lesser extent for rape fed cows. Rapeseed contains glucosinolates, which, even at low levels, have been shown to suppress thyroid function and reduce iodine in milk^41,42^. Additionally, Givens, *et al*.^43^ found less selenium in rape meal than in wheat and soybean meal. Whilst the content of antioxidants and trace elements in milk from the control and rapeseed groups followed similar patterns during the trial, feeding rape does appear to have a slightly suppressive effect on all these constituents - excepting that milk from these cows was slightly but significantly lower in manganese, β carotene and zeaxanthin before the trial started. Given this effect, care should be taken with supplementation to ensure cows (and consumers) are not deficient if dairy farms replace soya bean meal with home-grown feeds, to enhance sustainability.

Our other novel finding from this study is that replacing wheat and barley with naked oats for 14 days dramatically increased iodine concentration in milk, especially for organic cows. Despite continuity in the rate of iodine supplementation of the conventional herd, milk iodine concentration increased by 22% for conventional cows, by 85% for the organic herd (Fig. 4) and by 40% and 42% in the non-supplemented control and rape fed cows respectively. Iodine in milk decreased again in the 14 days after oat feeding was withdrawn (between date D and E) - this pattern was not seen for other trace element or antioxidants. For all but 1 of the 80 organic cows, milk iodine concentration increased between date C (on wheat) and D (after feeding oats) and declined when diets changed back to wheat (date E). In comparable figures for the conventional herd, 77(out of the 80) cows showed an increase in milk iodine between dates C to D, then for 75, this fell again between D and E samples. The mixed diets with oats were only marginally higher in iodine compared with those using wheat (1.80 v 1.66 mg/kg DM). We found no other studies reporting a direct or indirect effect of wheat or naked oats on iodine metabolism. The explanation is unclear but warrants further investigation - potentially offering an acceptable method to enhance iodine supply to organic cows and consumers.

## Conclusions

We set out to understand the influence of different aspects of dairy feeding on milk quality with respect to consumers’ health. However, although findings do confirm previous knowledge in many respects, the quest for explanations proved challenging. Changes in diets, blood and milk composition from the same apparent interventions against contrasting feeding systems, with comparable cows, raised a number of questions, as well as novel findings.

There is confirmation that organic management produced milk higher in many beneficial PUFA compared to conventional milk, especially n-3. However, if circulating n-3 levels in plasma reflect their absorption, this does not appear to be a direct response of the cows either to more n-3 supplied in the organic diets or reduced hydrogenation of dietary PUFA from feeding red and white clover silage. A similar picture, relating to lipid transfer from diet into milk, emerged from adding full-fat rapeseeds to the diets. Intake and circulating levels of n-3 were lower yet n-3 output in milk was higher than for control cows.

Whilst organic management and feeding rape improved the fatty acid profile, in the absence of mineral and vitamin fortification of silage diets, both strategies reduced concentrations of many vitamins, antioxidants and trace elements in milk. Over the last few years the organic sector has addressed trace element deficiencies, which could have altered the mineral profile of commercial organic milk. However, milk from oilseed supplementation of housed cows or from organic cows on winter feeding, could be vulnerable to oxidation if diets are not also fortified by vitamins or antioxidants, especially relevant due to the elevated PUFA content of this milk.

Under the circumstance of this study, feeding naked oats marginally increased iodine intake but caused a much more substantial increase to the iodine content of milk, especially in the organic herd in the absence of supplementation. This could be a natural approach to maintain iodine supply to consumers but care will be needed to avoid dangerously high concentrations in milk, if mineral supplementation is also offered.

## Supplementary information

Supplementary tables
