# Supplementary material for: Differing responses in milk composition from introducing rapeseed and naked oats to conventional and organic dairy diets
Source: Sci Rep. 2019 May 31;9:8115. doi: 10.1038/s41598-019-44567-8 (PMC6544624; doi:10.1038/s41598-019-44567-8)
Supplement: Supplementary file 1 — Supplementary tables [file 41598_2019_44567_MOESM1_ESM.pdf]

Differing responses in milk composition from introducing rapeseed and naked oats to conventional and organic dairy diets.

Gillian Butler[1]*, Sokratis Stergiadis[2], Eleni Chatzidimitriou[1], Enrica Franceschin[3], Hannah R Davis[1], Carlo Leifert[4] and Håvard Steinshamn[5]

[1] School of Natural and Environmental Science, Newcastle University, Newcastle upon Tyne NE1 7RU, UK
[2] University of Reading School of Agriculture, Policy and Development, Earley Gate, Reading RG6 6AR, UK
[3] Mérieux NutriSciences, Via Fratta, 25, 31023 Resana TV, Italy
[4] Centre for Organics Research, Southern Cross University, Lismore, NSW, Australia
[5] Norwegian Institute of Bioeconomy Research, Department of Grassland and Livestock, Norway

* corresponding author: gillian.butler@ncl.ac.uk

Supplementary tables:

Key for all tables:

- ANOVA p-values: ***= p < 0.001, **= p< 0.01; *= p< 0.05, t= 0.1>p <0.05, ns= p > 0.1.
- Conven= conventional management, sem= standard errors of means
- Fatty acids: VA= vaccenic acid (C18:1, t11), OA= oleic acid (C18:1 c9), LA = linolenic acid (C18:2, n=6), GLA = γ-linolenic acid, ALA = α-linolenic acid (C18:3, n-3), CLA9 = conjugated linoleic acid (C18:2c9t11), ukCLA, ukCLA2, ukCLA3 & ukCLA6 = unknown conjugated linoleic acid isomers, EPA = eicosapentaenoic acid (c20:5, n-3), DPA = docosapentaenoic acid (C22:5, n-3), DHA = docosahexaenoic acid (C22:6, n-3), SFA= saturated fatty acids, MUFA=monounsaturated fatty acids, PUFA=polyunsaturated fatty acids, n-3= omega-3 fatty acids, n-6=omega-6 fatty acids

Table S1 Mean milk yield and basic quality prior to the trial (date A) for management and treatment groups

	Management (M)		Supplement (S)			ANOVA p-values		
	Conven	Organic	control	rape	sem	M	S	MxS
	n=40	n=39	n=39	n=40	n=79			
Days since calving	114	145	129	131	8.0	t	ns	ns
Milk yield (Kg/cow/day)	27.7	27.7	27.3	28.1	0.76	ns	ns	ns
Fat %	4.37	4.05	4.21	4.22	0.060	**	ns	ns
Protein %	3.04	3.21	3.10	3.15	0.039	*	ns	ns
Lactose %	4.50	4.50	4.49	4.51	0.005	ns	ns	ns
Fat yield (kg/cow/day)	1.21	1.12	1.15	1.18	0.037	ns	ns	ns
Protein yield (kg/cow/day)	0.82	0.89	0.84	0.88	0.022	ns	ns	ns
Energy corrected milk yield (kg/cow/day)	30.9	30.2	30.0	31.1	0.85	ns	ns	ns

Table S2. Chemical composition of premixes, total mixed rations (TMR) and parlour cake; means (standard errors of means in parenthesis)

	Conventional					Organic				
	Control		Rape		Parlour cake.[1]	Control		Rape		Parlour cake [1]
	Premix	TMR	Premix	TMR		Premix	TMR	Premix	TMR	
Period 1 and 3, n=3										
DM, g/kg	766 (5.7)	334 (4.4)	795 (5.4)	318 (13)	866 (3.1)	773 (2.6)	291 (16)	831 (6.1)	309 (19)	877 (0.7)
Ash	62 (3.9)	83 (3.6)	66 (4.1)	84 (2.3)	74(3.8)	39 (0.8)	107 (13)	65 (15)	108 (14)	105 (1.5)
CP	258 (7.7)	171 (18)	235 (3.7)	153(1.0)	195 (2.6)	213 (3.7)	180 (6.2)	171 (5.2)	178 (10)	189 (0.7)
Fat	17 (2.3)	65 (6.7)	154 (24)	104 (9.9)	57 (3.3)	18 (1.2)	51 (3.1)	165 (9.8)	85 (4.7)	52 (1.2)
Starch	NA	51 (10)	NA	57 (22)	136 (4.0)	NA	136 (45)	NA	101 (32)	204 (6.2)
NDF	148 (12)	447 (5.7)	244 (21)	464 (12)	355 (2.3)	162 (20)	445 (10)	229 (19)	444 (9.5)	324 (0.3)
ADF	102 (12)	306 (11)	144 (5.8)	309 (4.1)	212 (12.5)	110 (16)	313 (7.1)	133 (11)	328 (0.9)	236 (1.3)
IVTD, %	NA	88 (2.4)	NA	88 (1.7)	85 (0.6)	NA	77 (1.5)	NA	80 (2.1)	82 (1.0)
dNDF, %	NA	72 (4.7)	NA	74 (3.2)	57 (1.2)	NA	48 (3.4)	NA	55 (4.5)	44 (3.1)
NEL, MJ/kg TS	7.4 (0.03)	6.7 (0.23)	9.5 (0.38)	7.4 (0.12)	7.3 (0.12)	7.6 (0.10)	6.2 (0.15)	9.8 (0.15)	6.8 (0.06)	7.0 (0.00)
Period 2, n=2										
DM, g/kg	752 (1.6)	315 (17)	785 (8.8)	320 (5.3)		756 (2.0)	328 (1.3)	817 (1.6)	335 (5.1)	
Ash	66 (0.3)	82 (8.3)	62 (1.0)	73 (3.4)		46 (1.2)	82 (1.1)	48 (6.3)	78 (4.6)	
CP	222 (3.5)	158 (5.5)	205 (6.0)	146 (3.5)		195 (3.0)	147 (0.5)	169 (12)	160 (2.0)	
Fat	38 (8.0)	83 (4.5)	136 (15)	115 (3.0)		41 (6.0)	42 (1.5)	152 (27)	70 (0.5)	
Starch	NA	72 (7.5)	NA	53 (4.5)		NA	234 (0.5)	NA	181 (22)	
NDF	131 (9.5)	456 (16)	194 (40)	457 (3.0)		144 (3.0)	410 (12)	209 (9.0)	433 (33)	
ADF	79 (7.5)	315 (9.5)	123 (17)	311 (7.0)		90 (5.5)	305 (24)	119 (7.5)	327 (14)	
IVTD, %	NA	87 (5.0)	NA	88 (1.0)		NA	75 (1.0)	NA	74 (1.0)	
dNDF, %	NA	72 (11)	NA	73 (2.0)		NA	39 (8.0)	NA	39 (8.0)	
NEL, MJ/kg TS	7.8 (0.2)	7.1 (0.05)	9.4 (0.4)	7.8 (0.07)		8.0 (0.15)	6.5 (0.15)	9.8 (0.4)	6.9 (0.15)	

[1]Parlour cake were the same in all three periods

Key to abbreviation: CP=crude protein, NDF= neutral detergent fibre, ADF= acid detergent fibre, IVTD= *in vitro* digestability, dNDF=fibre digestibility , NEL=net nenergy for lactation, NA=not available

Table S3 Near infra-red spectrophotometry (NIR) prediction of silage quality, mean values (standard errors of mean in parenthesis) for 3 samples of each type collected over the duration of the trial

Variable	units	1st cut Conventional (n=3)	2nd cut Organic (n=3)	3rd cut Organic (n=3)
Dry matter	%	30.5 (1.7)	27.6 (0.5)	24.9 (1.7)
D- value		68 (0.6)	62 (0.6)	68 (0.6)
ME	MJ/Kg DM	10.9 (0.09)	9.9 (0.12)	10.9 (0.06)
FME	MJ/Kg DM	9.0 (0.03)	7.6 (0.10)	8.4 (0.09)
CP	% DM	13.1 (0.20)	15.3 (0.12)	16.7 (0.32)
pH		3.9 (0.12)	4.3 0.03)	4.0 (0.06)
amN	% total N	5.4 (0.9)	11.3 (0.2)	9.9 (0.3)
ADF	% DM	30.4 (0.5)	30.1 (0.1)	27.7 (0.2)
NDF	% DM	45.9 (0.9)	44.9 (0.2)	41.6 (0.5)
ash	% DM	8.1 (0.19)	9.2 (0.07)	9.2 (0.03)
TFA	% DM	11.4 (0.5)	11.2 (0.4)	13.3 (0.2)
LA	% DM	10.5 (0.7)	7.9 (0.5)	10.8 (0.5)
AA	% DM	0.6 (0.06)	2.5 (0.04)	2.1 (0.25)
BA	% DM	0.15 (0.11)	0.19 (0.08)	0.01 (0.01)
Estimated intakel	gDM/kgW^0.75	97 (2.3)	97 (0.6)	97 (1.8)

Key to abbreviations: ME=metabolisable energy for ruminants, FME=fermentable metabolisable energy, CP=crude protein, amN= ammonia nitrogen, ADF=acid detergent fibre, NDF=neutral detergent fibreTFA=total fermentation acids, LA=lactic acids, AA=acetic acid, BA=butyric acid

Table S4 Mean concentrations of a) trace elements (μg/l) and b) antioxidants (μg/ml) in milk prior to the trial (date A) for management and treatment groups

	Management		Supplement			ANOVA p-values		
	Conven	Organic	control	rape	sem	M	S	MxS
a)Trace elements	n=40	n=38	n=40	n=38	n=78			
copper	51	42	45	48	2.4	t	ns	ns
zinc	3503	3550	3525	3527	83	ns	ns	ns
managenese	31	26	33	24	1.3	*	***	ns
selenium	24.3	14.3	19.2	19.8	0.64	***	ns	ns
iodine	181	56	126	113	8.3	***	t	*
molybdenum	40	59	49	49	1.7	***	ns	ns
b) Antioxidants	n=40	n=33	n=39	n=34	n=73			
α-tocopherol	0.56	0.38	0.48	0.48	0.018	***	ns	t
retinol	0.32	0.22	0.28	0.27	0.008	***	t	ns
β-carotene	0.18	0.15	0.18	0.15	0.006	*	*	*
total carotenoids	0.18	0.15	0.18	0.16	0.006	*	*	*
lutein	2.4	2.6	2.8	2.2	0.11	ns	**	ns
zeaxanthin	1.14	0.33	0.82	0.73	0.062	***	*	ns

Table S5. Key fatty acids and fatty acid groups (g/kg total fatty acid) in forages and novel feed ingredients (mean values and standard errors of means in parenthesis, n=3)

	1st cut conventional silage	2nd cut organic silage	3rd cut organic silage	Organic Wholecrop wheat	Naked-oat grain	Rapeseeds
C12:0	2.8 (0.37)	2.1 (0.20)	1.9 (0.16)	1.8 (0.17)	1.1 (0.82)	0.1 (0.01)
C14:0	6.4 (0.49)	5.2 (0.11)	4.4 (0.10)	16.1 (0.24)	2.1 (0.11)	0.04 (0.12)
PA	174 (1.7)	190 (3.5)	184 (6.7)	221 (1.8)	154 (2.9)	45 (0.1)
OA	29 (2.4)	19 (2.2)	18 (0.7)	86 (5.9)	337 (2.4)	575 (0.9)
LA	151 (2.4)	168 (6.0)	162 (3.6)	371 (11.2)	422 (2.3)	195 (0.5)
ALA	537 (8.6)	478 (15.9)	508 (14.8)	186 (11.2)	25.1 (2.4)	105 (1.0)
SFA	240 (2.9)	286 (10.2)	268 (13.5)	312 (7.3)	176 (4.6)	71 (0.2)
MUFA	51 (4.8)	47 (4.3)	41 (1.2)	113 (5.9)	355 (2.9)	617 (1.6)
PUFA	709 (7.0)	667 (14.4)	691 (14.3)	575 (10.4)	469 (6.9)	312 (1.5)
n-3	551 (8.7)	490 (15.9)	521 (14.3)	196 (10.5)	43.8 (6.8)	114 (1.2)
n-6	156 (2.3)	174 (6.3)	167 (3.4)	374 (10.8)	424 (2.3)	197 (0.4)
n-6:n-3	0.28 (0.008)	0.36 (0.021)	0.32 (0.011)	1.9 (0.14)	10.2 (1.85)	1.7 (0.01)

Table S6. Concentrations of key fatty acids and fatty acid groups in premixes, total mixed rations (TMR) and parlour cake (mean values in g/kg total fatty acid with standard errors of means in parenthesis)

	Conventional					Organic				
	Control		Rape		Parlour cake [1]	Control		Rape		Parlour cake [1]
	Premix	TMR	Premix	TMR		Premix	TMR	Premix	TMR	
**Period 1 and 3**	n=3	n=3	n=3	n=3	n=3	n=3	n=3	n=3	n=3	n=3
C12:0	0.72 (0.13)	4.7 (2.2)	0.25(0.21)	1.2 (0.04)	165 (1.7)	0.36 (0.05)	2.1 (0.16)	0.2 (0.05)	1.8 (1.2)	15.2 (0.5)
C14:0	1.3 (0.06)	6.0 (0.25)	0.56 (0.06)	3.3 (0.38)	58.6 (0.8)	1.5 (0.03)	5.6 (0.81)	0.56 (0.01)	3.0 (0.85)	6.4 (0.20)
PA	162 (13)	165 (5.4)	54 (0.9)	103 (2.2)	150 (2.1)	163 (1.9)	192 (2.9)	56.6 (1.4)	93.8 (9.0)	113 (2.6)
OA	259 (27)	56.5 (14)	554 (4.5)	328 (9.4)	233 (2.3)	243 (2.2)	55.4 (18)	576 (19)	403 (32)	252 (5.2)
LA	454 (19)	173 (8.2)	215 (4.0)	176 (1.0)	242 (9.5)	482 (4.0)	214 (16)	199 (15)	205 (4.5)	517 (5.3)
ALA	43.6 (7.1)	499 (14)	94.5 (2.6)	290 (4.2)	32.9 (2.1)	39.4 (2.1)	406 (27)	81.4 (14)	195 (18)	17.0 (0.2)
SFA	213 (16)	229 (6.2)	83.3 (0.8)	152 (7.0)	447 (5.2)	206 (2.9)	278 (7.1)	89.4 (5.8)	144 (15)	190 (4.4)
MUFA	278 (8.0)	80.5 (12)	593 (4.9)	364 (9.1)	272 (4.3)	259 (2.0)	80.8 (18)	617 (22)	442 (32)	269 (5.1)
PUFA	509 (12)	691 (6.3)	323 (4.1)	484 (3.0)	280 (11)	535 (4.8)	641 (11)	294 (28)	414 (19)	541 (5.4)
n-3	51.3 (6.7)	511 (14)	105 (0.5)	303 (4.1)	36.4 (1.4)	49.0 (3.7)	445 (8.6)	91.2 (14)	205 (16)	21.8 (0.4)
n-6	456 (19)	177 (8.0)	217 (3.9)	179 (1.1)	243 (9.5)	485 (4.0)	220 (16)	201 (15)	208 (5.6)	518 (5.4)
n-6:n-3	9.3 (1.4)	0.35 (0.03)	2.1 (0.03)	0.59 (0.03)	6.7 (0.08)	10.0 (0.75)	0.46 (0.03)	2.26 (0.18)	1.03 (0.08)	23.8 (0.5)
**Period 2**	n=2	n=2	n=2	n=2		n=2	n=2	n=2	n=2	
C12:0	0.32 (0.03)	7.7 (1.9)	0.14 (0.03)	6.2 (0.39)		0.38 (0.18)	7.8 (0.48)	0.16 (0.10)	3.3 (1.1)	
C14:0	1.8 (0.02)	7.0 (0.7)	0.68 (0.02)	4.3 (0.89)		1.7 (0.19)	6.8 (1.8)	0.72 (0.19)	3.5 (0.74)	
PA	160 (2.3)	157 (4.1)	62.8 (1.7)	106 (3.9)		147 (12)	198 (8.4)	64.7 (5.5)	112 (3.0)	
OA	326 (3.8)	86.9 (3.5)	545 (4.1)	310 (18)		356 (30)	122 (4.7)	533 (8.5)	357 (12)	
LA	431 (8.3)	206 (0.7)	233 (1.1)	191 (1.4)		400 (27)	301 (7.7)	235 (12)	232 (3.6)	
ALA	255 (0.2)	444 (9.0)	82.3 (0.6)	298 (16)		30.0 (6.0)	231 (8.4)	87.9 (12)	184 (12)	
SFA	187 (3.3)	220 (9.1)	90.9 (3.4)	154 (5.5)		178 (17)	296 (21)	91.4 (5.9)	175 (3.9)	
MUFA	342 (2.1)	110 (3.2)	583 (1.9)	342 (19)		375 (34)	145 (5.7)	572 (6.4)	393 (11)	
PUFA	472 (5.4)	670 (10)	326 (1.4)	504 (15)		447 (20)	558 (15)	336 (0.5)	433 (8.1)	
n-3	38.3 (3.0)	455 (9.3)	93.0 (0.4)	309 (16)		44.2 (7.0)	250 (8.1)	98.2 (12)	197 (11)	
n-6	433 (8.1)	213 (1.4)	232 (1.1)	194 (1.3)		402 (27)	305 (8.0)	237 (12)	234 (3.6)	
n-6:n-3	11.4 (1.1)	0.47 (0.01)	2.5 (0.00)	0.63 (0.03)		9.4 (2.1)	1.22 (0.02)	2.5 (0.41)	1.22 (0.09)	

[1] Parlour cakes were the same in all three periods

Table S7a. Mean concentrations of individual saturated fatty acid (g/kg total fatty acid) in milk prior to the trial (date A) for management and treatment groups

	Management		Supplement			ANOVA p-values		
Fatty acid	Conven	Organic	control	rape	sem	M	S	MxS
	n=40	n=38	n=39	n=39	n=78			
C4:0	24.1	32.6	28.8	27.7	0.58	***	t	ns
C5:0	0.27	0.29	0.3	0.27	0.008	ns	t	ns
C6:0	20.5	25.0	23.0	22.4	0.35	***	ns	ns
C7:0	0.28	0.25	0.28	0.26	0.011	t	ns	ns
C8:0	11.3	13.1	12.3	12.1	0.19	***	ns	ns
C9:0	0.32	0.37	0.31	0.39	0.049	ns	ns	ns
C10:0	26.2	29.0	27.7	27.4	0.56	*	ns	ns
C11:0	2.29	2.69	2.54	2.43	0.074	**	ns	ns
C12:0	37.2	36.1	36.8	36.5	0.78	ns	ns	ns
C13:0	1.74	1.66	1.76	1.64	0.056	ns	ns	ns
C14:0	120	121	122	120	1.7	ns	ns	ns
C15:0	12.6	12.5	12.7	12.4	0.24	ns	ns	ns
C16:0	357	336	351	343	4.5	*	ns	ns
C17:0	5.4	6.0	5.6	5.7	0.077	***	ns	ns
C18:0	92	94	91	96	1.9	ns	ns	ns
C20:0	1.34	1.45	1.38	1.41	0.022	*	ns	ns
C22:0	0.61	0.75	0.67	0.68	0.015	***	ns	ns
C23:0	0.21	0.32	0.26	0.26	0.009	***	ns	ns
C24:0	0.44	0.52	0.48	0.48	0.011	***	ns	ns

Table S7b. Mean concentrations of individual monounsaturated fatty acid (g/kg total fatty acid) in milk prior to the trial (date A) for management and treatment groups

	Management		Supplement			ANOVA p-values		
Fatty acid	Conven	Organic	control	rape	sem	M	S	MxS
	n=40	n=38	n=39	n=39	n=78			
c9C14:1	9.5	9.2	9.6	9.1	0.32	ns	ns	ns
c9C15:1	0.13	0.12	0.13	0.12	0.005	ns	ns	*
t9C16:1	3.3	3.3	3.2	3.3	0.05	ns	ns	ns
c9C16:1	19.8	16.9	18.5	18.4	0.40	***	ns	t
c9C17:1	2.5	2.1	2.3	2.3	0.08	*	ns	ns
t6+t7+t8C18:1	1.52	1.73	1.62	1.63	0.025	***	ns	t
t9C18:1	1.18	1.28	1.23	1.23	0.019	**	ns	ns
t10C18:1	1.53	2.10	1.82	1.80	0.046	***	ns	ns
VA	8.6	10.7	9.5	9.8	0.22	***	ns	ns
t12-14C18:1	2.6	2.8	2.7	2.6	0.06	ns	ns	t
OA	187	172	176	184	4.9	ns	ns	ns
t15C18:1	2.4	2.8	2.5	2.6	0.05	***	ns	ns
c1C18:1	4.9	3.7	4.2	4.5	0.21	**	ns	ns
c12C18:1	0.87	1.60	1.23	1.23	0.048	***	ns	ns
c13C18:1	0.72	0.60	0.68	0.64	0.039	ns	ns	ns
c14+t16C18:1	2.7	3.4	3.1	3.0	0.06	***	ns	ns
c15C18:1	1.17	1.15	1.16	1.16	0.018	ns	ns	ns
C19:1	0.95	1.12	1.04	1.02	0.02	***	ns	t
c8C20:1	0.87	0.97	0.93	0.90	0.019	**	ns	*
C22:1	0.09	0.13	0.11	0.11	0.003	***	ns	t
C24:1	0.10	0.09	0.10	0.10	0.003	**	ns	ns

Table S7c. Mean concentrations of individual polyunsaturated fatty acid (g/kg total fatty acid) in milk prior to the trial (date A) for management and treatment groups

	Management		Supplement			ANOVA p-values		
Fatty acid	Conven	Organic	control	rape	sem	M	S	MxS
	n=40	n=38	n=39	n=39	n=78			
C18:2n3	0.52	0.57	0.54	0.55	0.011	*	ns	t
t10t14C18:2	0.23	0.24	0.24	0.23	0.006	ns	ns	ns
c9t13C18:2	0.47	0.78	0.60	0.64	0.024	***	ns	t
t9,12C18:2n6	0.56	0.71	0.64	0.64	0.018	***	ns	t
t8c13C18:2	0.85	1.03	0.95	0.93	0.026	***	ns	ns
c9t12C18:2	0.93	1.04	1.01	0.95	0.022	*	ns	ns
t9c12C18:2	0.42	0.54	0.49	0.47	0.011	***	ns	ns
cis/trans	0.19	0.20	0.20	0.19	0.006	ns	ns	**
t11c15C18:2	1.90	1.87	1.86	1.91	0.036	ns	ns	ns
LA	9.5	17.5	13.2	13.6	0.53	***	ns	ns
uk3C18:2	1.00	1.04	1.02	1.02	0.013	ns	ns	ns
uk2C18:2	0.68	0.51	0.61	0.58	0.013	***	ns	ns
c9c15C18:2	0.31	0.32	0.33	0.3	0.007	ns	*	ns
GLA	0.13	0.18	0.15	0.16	0.005	***	ns	ns
ALA	5.0	9.9	7.5	7.3	0.30	***	ns	ns
CLA9	4.2	5.1	4.6	4.6	0.11	***	ns	ns
ukCLA1	0.52	0.57	0.53	0.55	0.011	*	ns	ns
ukCLA2	0.28	0.25	0.27	0.26	0.006	*	ns	ns
ukCLA3	0.43	0.36	0.40	0.39	0.008	***	ns	ns
ukCLA6	0.13	0.18	0.15	0.16	0.005	***	ns	ns
c9,13,15C18:3	0.17	0.15	0.16	0.16	0.004	*	ns	ns
C20:2n6	0.23	0.39	0.31	0.30	0.011	***	ns	ns
c9,15t11cC18:3	0.37	0.32	0.34	0.35	0.009	*	ns	ns
C20:3n6	0.49	0.78	0.61	0.65	0.026	***	ns	ns
C20:3	0.11	0.20	0.15	0.15	0.006	***	ns	ns
C20:4	0.70	0.97	0.82	0.85	0.022	***	ns	ns
C23:0	0.21	0.32	0.26	0.26	0.009	***	ns	ns
C22:2	0.60	0.34	0.46	0.47	0.017	***	ns	ns
EPA	0.59	0.67	0.63	0.63	0.013	**	ns	t
C22:3n3	0.08	0.07	0.07	0.08	0.003	*	ns	ns
C22:4	0.14	0.21	0.18	0.18	0.007	***	ns	ns
DPA	0.96	1.11	1.00	1.06	0.024	**	ns	ns
DHA	0.09	0.08	0.09	0.09	0.003	ns	ns	ns

Table S7d. Mean concentrations of fatty acid groups (g/kg total fatty acid) in milk prior to the trial (date A) for management and treatment groups

	Management		Supplement			ANOVA p-values		
Fatty acid	Conven	Organic	control	rape	sem	M	S	MxS
	n=40	n=38	n=39	n=39	n=78			
SFA	715	714	719	710	5.5	ns	ns	ns
MUFA	252	238	241	249	5.3	ns	ns	ns
PUFA	32.8	48.2	40.1	40.4	1.01	***	ns	ns
n3	1.01	1.53	1.27	1.26	0.034	***	ns	ns
n6	1.37	2.27	1.79	1.83	0.059	***	ns	ns
n3:n6	0.74	0.68	0.73	0.70	0.010	***	ns	ns
n6:n3	1.35	1.49	1.40	1.44	0.020	***	ns	ns
Oddchain FA	25.7	26.3	26.2	25.8	0.30	ns	ns	ns
Long chain n-3	1.73	1.93	1.80	1.86	0.035	**	ns	ns

Table S8. Mean milk yield and basic quality during the trial (dates B-E), for management and treatment groups

	Management (M)		Supplement (S)			ANOVA p-values		
	Conven	Organic	control	rape	sem	M	S	MxS
	n=160	n=160	n=160	n=160	n=320			
Milk yield (Kg/cow/day)	27.0	27.4	26.2	28.2	0.35	ns	ns	ns
Fat %	4.13	3.88	4.05	3.96	0.033	*	ns	ns
Protein %	3.16	3.12	3.22	3.06	0.019	ns	*	ns
Lactose %	4.47	4.43	4.38	4.52	0.014	ns	**	ns
Fat yield (kg/cow/day)	1.10	1.06	1.06	1.11	0.015	ns	ns	t
Protein yield (kg/cow/day)	0.84	0.85	0.83	0.86	0.010	ns	ns	ns
Energy corrected milk yield (kg/cow/day)	29.5	29.1	28.5	30.1	0.36	ns	ns	ns

Table S9. Mean metabolic profile from blood taken at date E (mean values in mmol per litre and sem), for management and treatment groups

	Management (M)		Supplement (S)			ANOVA p-values		
	Conven	Organic	control	rape	sem	M	S	MxS
	n=40	n=40	n=40	n=40	n=80			
Albumin	35	35	35	35	0.3	ns	ns	ns
Globulin	58	57	57	58	1.1	ns	ns	ns
Total protein	92	92	92	92	1.0	ns	ns	ns
GGT	42	38	39	40	1.6	ns	ns	ns
Urea	4.8	5.2	4.9	5.1	0.11	*	ns	*
BHB	0.61	0.59	0.57	0.63	0.028	ns	ns	ns
Magnesium	1.00	1.04	1.02	1.02	0.009	t	ns	**
Phosphate	1.88	2.06	2.00	1.95	0.025	***	ns	ns

Key to abbreviations:GGT= Gamma glutamyltransferase, BHB=beta hydroxibutyrate

Table S10. Mean concentrations of a) trace elements (µg/l) and b) antioxidants (µg/ml) in milk during the trial (dates B-E) for management and treatment groups

	Management		Supplement			ANOVA p-values		
a)Trace elements	Conven	Organic	control	rape	sem	M	S	MxS
	n=160	n=160	n=160	n=160	n=320			
copper	44.8	36.3	41.7	39.4	1.00	*	ns	ns
zinc	3065	3109	3090	3084	35.8	ns	ns	ns
managenes	29. 6	25.4	28.7	26.2	0.59	*	ns	ns
selenium	27.3	15.4	22.2	20.5	0.38	***	**	ns
iodine	287	152	237	201	6.1	***	*	ns
molybdenum	42.9	50.1	48.8	44.2	0.66	**	*	ns
b) Antioxidants	n=156	n=155	n=155	n=156	n=311			
a-tocopherol	0.60	0.29	0.46	0.43	0.011	***	ns	ns
retinol	0.29	0.19	0.26	0.23	0.004	***	***	ns
b-carotene	0.19	0.14	0.20	0.13	0.004	***	***	ns
total carotenoids	0.19	0.14	0.20	0.13	0.004	***	***	ns
lutein	2.21	2.62	2.51	2.32	0.054	*	ns	ns
zeaxanthin	1.28	0.32	0.86	0.74	0.035	***	ns	ns

Table S11a. Mean concentrations of individual saturated fatty acids (g/kg total fatty acid) in milk during the trial (dates B-E) for management and treatment groups

	Management		Supplement			ANOVA p-values		
	Conven	Organic	control	rape	sem	M	S	MxS
	n=160	n=159	n=159	n=160	n=319			
C4:0	26.7	32.2	28.8	30.1	0.30	***	*	***
C5:0	0.29	0.27	0.31	0.25	0.004	ns	***	t
C6:0	21.2	23.6	23.7	21.1	0.18	***	***	ns
C7:0	0.27	0.23	0.31	0.20	0.006	**	***	ns
C8:0	11.7	12.1	13.2	10.6	0.11	ns	***	ns
C9:0	0.32	0.25	0.36	0.21	0.007	***	***	ns
C10:0	25.7	26.2	30.6	21.3	0.34	ns	***	ns
C11:0	2.3	2.3	2.7	1.9	0.03	ns	***	ns
C12:0	35.5	30.3	39.3	26.5	0.47	***	***	ns
C13:0	1.64	1.44	1.91	1.16	0.029	**	***	ns
C14:0	112	107	123	95.7	0.96	**	***	ns
C15:0	11.0	10.8	12.5	9.2	0.11	ns	***	ns
C16:0	301	281	346	236	3.5	***	***	ns
C17:0	4.4	5.1	5.3	4.2	0.05	***	***	ns
C18:0	120	127	92.4	155	2.0	t	***	t
C20:0	1.94	2.16	1.53	2.57	0.033	***	***	ns
C22:0	0.64	0.84	0.71	0.77	0.009	***	**	ns
C23:0	0.16	0.27	0.25	0.18	0.004	***	***	ns
C24:0	0.36	0.46	0.47	0.35	0.006	***	***	ns

Table S11b. Mean concentrations of individual monounsaturated fatty acids (g/kg total fatty acid) in milk during the trial (dates B-E) for management and treatment groups

	Management		Supplement			ANOVA p-values		
	Conven	Organic	control	rape	sem	M	S	MxS
	n=160	n=159	n=159	n=160	n=319			
c9C14:1	9.1	7.8	9.8	7.1	0.15	**	***	ns
c9C15:1	0.12	0.12	0.14	0.10	0.003	ns	***	ns
t9C16:1	3.01	2.80	3.01	2.80	0.019	***	***	ns
c9C16:1	16.0	13.5	17.2	12.4	0.21	***	***	ns
c9C17:1	1.77	1.67	1.94	1.51	0.024	ns	***	ns
t6+t7+t8C18:1	3.6	4.3	1.8	6.0	0.13	***	***	**
t9C18:1	2.7	3.0	1.4	4.2	0.09	***	***	ns
t10C18:1	3.4	4.1	2.2	5.2	0.10	***	***	ns
VA	10.1	12.0	9.0	13.1	0.18	***	***	ns
t12-14C18:1	4.5	4.8	3.2	6.1	0.10	*	***	ns
OA	213	211	170	254	2.7	ns	***	ns
t15C18:1	3.6	4.1	2.6	5.0	0.08	***	***	*
c1C18:1	4.9	4.5	3.9	5.5	0.07	t	***	ns
c12C18:1	1.8	3.0	1.7	3.1	0.06	***	***	*
c13C18:1	0.66	0.59	0.56	0.69	0.012	*	**	ns
c14+t16C18:1	4.4	4.9	3.4	6.0	0.084	***	***	ns
c15C18:1	1.40	1.32	1.13	1.59	0.017	*	***	*
C19:1	1.17	1.25	1.07	1.35	0.015	*	***	ns
c8C20:1	1.30	1.41	1.08	1.63	0.019	*	***	ns
C22:1	0.21	0.27	0.14	0.34	0.007	***	***	ns
C24:1	0.128	0.129	0.098	0.159	0.0024	ns	***	ns

Table S11c. Mean concentrations of individual polyunsaturated fatty acids (g/kg total fatty acid) in milk during the trial (dates B-E) for management and treatment groups

	Management		Supplement			ANOVA p-values		
	Conven	Organic	control	rape	sem	M	S	MxS
	n=160	n=159	n=159	n=160	n=319			
C18:2n3	0.49	0.56	0.56	0.49	0.006	***	***	ns
t10t14C18:2	0.26	0.22	0.21	0.27	0.004	***	***	t
c9t13C18:2	0.60	1.15	1.03	0.72	0.027	***	***	***
t9,12C18:2n6	0.98	1.05	0.76	1.27	0.019	t	***	ns
t8c13C18:2	1.37	1.44	1.01	1.80	0.030	ns	***	ns
c9t12C18:2	1.33	1.41	1.05	1.69	0.024	ns	***	ns
t9c12C18:2	0.62	0.74	0.51	0.85	0.012	***	***	ns
cis/trans	0.24	0.29	0.22	0.31	0.004	***	***	**
t11c15C18:2	1.65	1.35	1.42	1.58	0.024	***	**	***
LA	10.5	18.9	14.8	14.6	0.27	***	ns	**
uk3C18:2	0.94	1.03	1.02	0.96	0.007	***	**	ns
uk2C18:2	0.54	0.38	0.51	0.41	0.006	***	***	ns
c9c15C18:2	0.35	0.38	0.29	0.44	0.006	*	***	*
GLA	0.15	0.21	0.2	0.16	0.004	***	***	ns
ALA	5.5	9.3	7.0	7.8	0.13	***	***	ns
CLA9	5.1	5.7	4.5	6.3	0.09	*	***	ns
ukCLA1	0.45	0.49	0.5	0.44	0.005	**	***	***
ukCLA2	0.34	0.27	0.26	0.35	0.005	***	***	ns
ukCLA3	0.41	0.31	0.35	0.37	0.005	***	ns	***
ukCLA6	0.18	0.23	0.19	0.22	0.003	***	**	ns
c9,13,15C18:3	0.17	0.12	0.14	0.16	0.003	***	***	*
C20:2n6	0.24	0.38	0.34	0.27	0.005	***	***	***
c9,15t11cC18:3	0.39	0.26	0.32	0.34	0.006	***	ns	*
C20:3n6	0.51	0.79	0.72	0.58	0.013	***	***	ns
C20:3	0.22	0.31	0.15	0.38	0.008	***	***	*
C20:4	0.64	0.91	0.86	0.69	0.012	***	***	***
C22:2	0.50	0.22	0.39	0.32	0.009	***	***	*
EPA	0.50	0.62	0.64	0.48	0.008	***	***	t
C22:3n3	0.08	0.067	0.085	0.062	0.0017	***	***	ns
C22:4	0.13	0.2	0.19	0.14	0.004	***	***	*
DPA	0.85	1.03	1.01	0.87	0.013	***	**	ns
DHA	0.08	0.08	0.08	0.08	0.002	ns	ns	t

Table S11d. Mean concentrations of groups of fatty acids (g/kg total fatty acid) in milk during the trial (dates B-E) for management and treatment groups

	Management		Supplement			ANOVA p-values		
	Conven	Organic	control	rape	sem	M	S	MxS
	n=160	n=159	n=159	n=160	n=319			
Calculated values								
SFA	677	663	723	617	3.6	*	***	ns
MUFA	287	287	235	338	3.3	ns	***	ns
PUFA	36.3	50.4	41.3	45.4	0.49	***	***	*
n3	10.3	14.1	11.7	12.7	0.14	***	***	ns
n6	15.7	24.8	19.8	20.6	0.30	***	ns	**
n3:n6	0.66	0.58	0.61	0.63	0.005	***	ns	**
n6:n3	1.52	1.79	1.69	1.62	0.017	***	t	***
Oddchain FA	22.2	22.4	25.7	18.9	0.22	ns	***	ns
Long chain n-3	1.73	2.11	1.96	1.88	0.019	***	ns	*

Table S12. Mean concentrations of a) trace elements trace elements (µg/l) and b) antioxidants (µg/ml) in milk for dates C and E (wheat) and date D when oats were fed

	Cereal			ANOVA p-value
	Wheat	Oats	sem	
a)Trace elements	n=160	n=80	n=240	
copper	39	40	1.10	ns
zinc	3093	3041	41.5	ns
manganese	27	28	0.63	ns
selenium	22	212	0.46	ns
iodine	214	277	6.4	***
molybdenum	45	45	0.66	ns
b) Anitoxidants	n=155	n=76	n=231	
α-tocopherol	0.44	0.43	0.014	ns
retinol	0.24	0.24	0.004	ns
β-carotene	0.17	0.16	0.004	**
total carotenoids	0.17	0.16	0.004	**
lutein	2.4	2.3	0.06	ns
zeaxanthin	0.79	0.81	0.040	ns

Table S13a. Mean concentrations of individual saturated fatty acids in milk for dates C and E (wheat) and date D when oats were fed

	Cereal			ANOVA p-value
	Wheat	Oats	sem	
	n=160	n=80	n=240	
C4:0	29.3	27.5	0.34	***
C5:0	0.27	0.27	0.004	ns
C6:0	22.0	22.1	0.21	ns
C7:0	0.24	0.26	0.006	***
C8:0	11.6	12.1	0.13	***
C9:0	0.27	0.30	0.008	***
C10:0	25.6	26.7	0.39	***
C11:0	2.25	2.36	0.038	***
C12	32.6	33.3	0.53	**
C13:0	1.52	1.59	0.032	**
C14:0	109	111	1.1	**
C15:0	10.8	10.9	0.13	ns
C16:0	286	296	40	***
C17:0	4.8	4.6	0.048	***
C18:0	127	120	2.3	***
C20:0	2.11	2.02	0.038	***
C22:0	0.77	0.75	0.010	*
C23:0	0.22	0.22	0.005	ns
C24:0	0.42	0.42	0.007	ns

Table S13b. Mean concentrations of individual monounsaturated fatty acids in milk for dates C and E (wheat) and date D when oats were fed

	Wheat	Oats	sem	ANOVA p-value
	n=160	n=80	n=240	
c9C14:1	8.3	8.7	0.17	***
c9C15:1	0.118	0.128	0.0031	*
t9C16:1	2.90	2.86	0.022	t
c9C16:1	14.4	15.1	0.23	***
c9C17:1	1.69	1.71	0.025	ns
t6+t7+t8C18:1	4.1	3.6	0.14	***
t9C18:1	2.9	2.7	0.10	***
t10C18:1	3.9	3.6	0.11	**
t11C18:1	11.4	10.0	0.20	***
t12+13+14C18:1	4.7	4.7	0.11	ns
c9C18:1	214	210	3.1	*
t15C18:1	3.9	3.7	0.09	***
c1C18:1	4.7	4.7	0.08	ns
c12C18:1	2.5	2.6	0.07	ns
c13C18:1	0.61	0.63	0.012	ns
c14+t16C18:1	4.8	4.5	0.10	***
c15C18:1	1.36	1.32	0.019	*
C19:1	1.24	1.19	0.017	*
c8C20:1	1.38	1.37	0.022	ns
C22:1	0.24	0.24	0.007	ns
C24:1	0.128	0.130	0.0028	ns

Table S13c. Mean concentrations of individual polyunsaturated fatty acids in milk for dates C and E (wheat) and date D when oats were fed

	Wheat	Oats	sem	ANOVA p-value
	n=160	n=80	n=240	
t11t15C18:2n3	0.54	0.54	0.007	ns
t10t14C18:2	0.24	0.21	0.005	***
c9t13C18:2	0.89	1.12	0.033	***
t9t12C18:2n6	1.03	1.04	0.023	ns
t8c13C18:2	1.44	1.41	0.035	ns
c9t12C18:2n6	1.40	1.38	0.029	ns
t9c12C18:2n6	0.70	0.68	0.013	ns
cis/trans	0.27	0.28	0.005	ns
t11c15C18:2n3	1.49	1.29	0.027	***
LAn6	15.0	15.2	0.32	ns
unknown3C18:2	1.01	0.97	0.008	***
unknown2C18:2	0.45	0.46	0.007	ns
c9c15C18:2n3	0.37	0.35	0.007	**
GLAn6	0.19	0.19	0.004	ns
ALNn3	7.6	6.7	0.13	***
CLA9	5.6	5.1	0.10	***
unknownCLA1	0.49	0.43	0.006	***
unknownCLA2	0.31	0.30	0.006	**
unknownCLA3	0.37	0.34	0.006	***
unknownCLA6	0.216	0.210	0.0035	ns
c9c13c15C18:3n3	0.150	0.125	0.0031	***
C20:2n6	0.31	0.32	0.006	ns
c9t11c15C18:3n3	0.33	0.30	0.007	***
C20:3n6	0.67	0.68	0.015	ns
C20:3n3	0.27	0.27	0.008	ns
C20:4n6	0.79	0.80	0.014	*
C22:2n6	0.35	0.37	0.011	***
EPAn3	0.56	0.57	0.009	ns
C22:3n3	0.075	0.076	0.0019	ns
C22:4n6	0.170	0.168	0.0040	ns
DPAn3	0.95	0.96	0.014	ns
DHAn3	0.083	0.082	0.0020	ns

Table S13d. Mean concentrations of fatty acid groups in milk for dates C and E (wheat) and date D when oats were fed

	Cereal			ANOVA p-value
	Wheat	Oats	sem	
	n=160	n=80	n=240	
SFA	667	673	4.0	**
MUFA	289	284	3.8	**
PUFA	44.2	42.9	0.6	***
n3	1.24	1.12	0.014	***
n6	2.06	2.09	0.035	ns
n3:n6	0.62	0.56	0.006	***
n6:n3	1.66	1.84	0.020	***
oddchain	22.1	22.4	0.24	*
Longchain n-3	1.93	1.95	0.022	ns
